# Predictive value of computed tomography with coronal reconstruction in right hemicolectomy with complete mesocolic excision for right colon cancers: a retrospective study

**DOI:** 10.1186/s12957-021-02307-1

**Published:** 2021-06-28

**Authors:** Hui Yu, Yong Zhuang, Jinliang Jian, Chunkang Yang

**Affiliations:** Department of Gastrointestinal Surgical Oncology, Fujian Cancer Hospital & Fujian Medical University Cancer Hospital, 420 Fuma Street, Jinan, Fuzhou, Fujian 350014 P.R. China

**Keywords:** Colonic neoplasms, Right hemicolectomy, Computed tomography, Vascular anatomy, Central vascular ligation, Complete mesocolic excision

## Abstract

**Background:**

Understanding the vascular anatomy is critical for performing central vascular ligation (CVL) in right hemicolectomy with complete mesocolic excision (CME). This study aimed to investigate the predictive value of multi-slice spiral computed tomography (MSCT) with coronal reconstruction in right hemicolectomy with CME.

**Methods:**

This is a retrospective descriptive study. Eighty patients with right colon cancer who underwent right hemicolectomy from December 2015 to January 2020 were included. The intraoperative reports (including imaging data) and MSCT images with coronal reconstruction were analysed and compared. The detection rates of the ileocolic vein (ICV) and ileocolic artery (ICA) roots and the accuracy in predicting their anatomical relationship were analysed. The detection rate and accuracy in predicting the location of the gastrocolic trunk of Henle (GTH), middle colic artery (MCA) and middle colic vein (MCV) were analysed. The distance from the ICV root to the GTH root (ICV-GTH distance) was measured and analysed. The maximum distance from the left side of the superior mesenteric artery (SMA) to the right side of the superior mesenteric vein (SMV), named the ‘lsSMA-rsSMV distance’, was also measured and analysed.

**Results:**

In seventy-four (92.5%) patients, both the ICV and ICA roots were located; their anatomical relationship was determined by MSCT, and the accuracy of the prediction was 97.2% (72/74). The GTH was located by MSCT in 75 (93.7%) patients, and the accuracy of the prediction was 97.33% (73/75). The MCA was located by MSCT in 47 (58.75%) patients, and the accuracy was 78.72% (37/47). The MCV was located by MSCT in 51 (63.75%) patients, and the accuracy of the prediction was 84.31% (43/51). The ICV-GTH distance was measured in 73 (91.2%) patients, and the mean distance was 4.28 ± 2.5 cm. The lsSMA-rsSMV distance was measured in 76 (95%) patients, and the mean distance was 2.21 ± 0.6 cm.

**Conclusions:**

With its satisfactory accuracy in predicting and visualising the information of key anatomical sites, MSCT with coronary reconstruction has some predictive value in CME with CVL in right hemicolectomy.

## Background

Since the concept of complete mesocolic excision (CME) was introduced for the surgical treatment of colon cancer, patients who undergo these procedures have achieved lower 5-year local recurrence rates (from 6.5 to 3.6%) and better 5-year cancer-related survival rates (from 82.1 to 89.1%) [1]. After promising results were reported, more centres began to adopt CME as a standard procedure for colon cancer surgery [2–4].

It is important to note that CME should be performed under the principle of central vascular ligation (CVL), which includes nearly full-length skeletonisation of the superior mesenteric vessels during right hemicolectomy, and this procedure was considered an extended dissection according to S. Toyota’s research [5] and the Japanese Society for Cancer of the Colon and Rectum (JSCCR) guidelines [6]. Compared to D3 resection, CME with CVL appears to facilitate the retrieval of longer specimens and more mesentery and nodal nodes, but the differences in long-term outcome between these procedures are still not clear [7].

Typically, right hemicolectomy can be performed through the following different approaches: cephalic approach, caudal approach, and central approach. Amongst these approaches, the central approach is usually considered the most consistent with the principle of radical tumour resection, which first requires dissection and ligation of the vessel roots in the superior mesenteric vascular region. However, due to the vascular variations in the superior mesenteric region [8–11], it is difficult for surgeons to expose the vessel roots and dissect lymph nodes in the CVL region during surgery. A lack of proper understanding of the vascular anatomy and central vascular ligation region might lead to intraoperative injury, bleeding and inadequate lymph node clearance and increases the difficulty of performing CME with CVL [12, 13].

Therefore, methods for predicting the critical anatomical sites of colon cancer before the operation have become an area of interest for surgeons [14, 15]. Surgeons and radiologists have tried to predict vascular anatomical variations with multidetector computed tomography (MDCT) angiography and three-dimensional CT (3D-CT), which have been reported to have high sensitivity, specificity, accuracy and reliability [16, 17].

However, preoperative MDCT angiography and 3D-CT are not commonly used, partly due to concerns of possible additional radiation exposure [18, 19], technical limitations and increased costs in some centres. More importantly, due to the lack of anatomical information apart from the blood vessels seen on angiographic images, these techniques cannot provide surgeons with an excellent visual prediction of the lymph node dissection regions, such as the range and size of the CVL region and its relationship with other anatomical structures, which is important in surgery [16, 17].

In this study, we attempted to use multi-slice spiral computed tomography (MSCT) with a coronal reconstruction technique to assess the vascular anatomy and CVL region that need to be explored during right hemicolectomy with CME.

## Materials and methods

This is a retrospective descriptive study. The study was approved by the ethics committee of Fujian Cancer Hospital & Fujian Medical University Cancer Hospital (ethical approval number: K2020-035-01). This study was carried out following the World Medical Association’s Code of Ethics (Helsinki Declaration).

The patient information, intraoperative reports and raw MSCT data of 80 patients with right colon cancer who underwent right hemicolectomy at Fujian Cancer Hospital & Fujian Medical University Cancer Hospital from December 2015 to January 2020 were collected from medical documents and databases.

### Inclusion and exclusion criteria

The inclusion criteria were as follows: right-sided colon cancer treated with right hemicolectomy; plain abdominal and pelvic and contrast-enhanced triple-phase MSCT scans performed before the operation, and original data that was available for reconstruction. The exclusion criterion was as follows: intraoperative reports (including imaging data) not available to assess the anatomy of the ileocolic vein (ICV), ileocolic artery (ICA), gastrocolic trunk of Henle (GTH), middle colic artery (MCA) and middle colic vein (MCV).

The details of the MSCT scan and coronal reconstruction technique are as follows: plain abdominal and pelvic and contrast-enhanced triple-phase MSCT scans were performed preoperatively with a high-speed 256-slice spiral CT scanner (Brilliance iCT, Philips Medical Systems (Cleveland) Inc., Cleveland, USA). After the patient practised deep breathing, a breath-hold scan ranging from the diaphragm to the lower edge of the pubic symphysis was performed with a 0.6 mm per slice thickness. The contrast agent used was ioversol (320 mg/mL, 1.2-1.5 mL/kg; injection flow rate, 3.0 mL/s). The arterial phase image was acquired 30 s after the injection. The portal phase image was acquired 70 s after the injection. The delayed phase image was acquired 240 s after the injection. The scanning parameters were as follows: tube voltage, 120 kV; automatic tube current and 0.5 s/r ball tube speed.

The original data of the portal phase scan were extracted and reconstructed in the coronal plane by multiplanar reconstruction (MPR) and maximum intensity projection (MIP) techniques. MSCT data coupled with the coronal reconstructed images were used to locate the roots of the ICV, ICA, GTH, MCA and MCV, the right side of the superior mesenteric vein (SMV) and the left side of the superior mesenteric artery (SMA) (with software from Philips InteliSpace Portal v4.0.4.10004, Phillips Healthcare Nederland B.V.).

### Analysis, measurements and calculations

The analysed data were compared with the intraoperative reports to evaluate accuracy. The anatomical relationship between the ICV and ICA was analysed on coronal and axial images with the software’s positioning function. The location of the GTH relative to the pancreas and ICV was compared with the intraoperative findings to determine the accuracy of the prediction. To gain further insight into the extent of the CVL region, we designed several measures. The straight-line distance from the ICV root to the GTH root (ICV-GTH distance) was measured, and the maximum distance from the left side of the SMA to the right side of the SMV (lsSMA-rsSMV distance) was also measured. Then, the means of the ICV-GTH distance and the lsSMA-rsSMV distance were analysed.

### Statistical analysis

Descriptive statistical methods were used. Statistical analysis was performed using SPSS 25.0 (IBM, Chicago). The accuracy of each prediction is represented as a rate. The distances are represented by the mean and standard deviation.

## Results

The patients ranged from 15 to 87 years of age and included 42 males and 38 females with BMIs ranging from 16.0 to 29.1; there were 10 caecum cancers, 46 ascending colon cancers, 14 hepatic flexure cancers and 10 proximal transverse colon cancers. All patients were operated on through the central approach. The ICV, ICA, GTH, MCV and MCA roots were exposed step-by-step during the operation to determine their positions and relative relationships, and these were recorded in the intraoperative report for comparisons (Table 1).

**Table 1:** Patient and tumour characteristics (*n* = 80)

	n (%)
Age median/range (years)	59 (15-87)
Sex	
Male	42 (52.5%)
Female	38 (47.5%)
BMI (mean ± SD, range)	22.5 ± 3.1 (16.0-29.1)
Tumour site	
Caecum	10 (12.5%)
Ascending colon	46 (57.5%)
Hepatic flexure	14 (17.5%)
Proximal transverse colon	10 (12.5%)
pT category^a^	
Tis	3 (3.75%)
T1	4 (5%)
T2	7 (8.75%)
T3	40 (50%)
T4	26 (32.5%)
pN category^a^	
pN0	37 (46.25%)
pN1	33 (41.25%)
pN2	10 (12.5%)
pM category^a^	
M0	69 (86.25%)
M1	11 (13.75%)
Stage	
Stage 0	3 (3.75%)
Stage I	8 (10%)
Stage II	23 (28.75%)
Stage III	35 (43.75%)
Stage IV	11 (13.75%)
Surgical approach	
D2 dissection	4 (5%)
CME	65 (81.25%)
Multivisceral resection	7 (8.75%)
Palliative operation	4 (5%)

^a^According to the 8th edition of the American Joint Committee on Cancer (AJCC) staging system [20]

The MSCT data of 80 patients were reconstructed and analysed as required. In 74 (92.5%) of the 80 patients, MSCT with coronal reconstruction was able to locate the ICV and ICA roots and determine their anatomical relationship, and these findings were confirmed by the intraoperative findings in 72 patients, so the accuracy was 97.2% (72/74). The location of the GTH relative to the pancreas was found in 75 (93.7%) patients, and the accuracy was 97.33% (73/75). The ICV-GTH distance was measured in 73 (91.2%) patients, and the mean distance was 4.28 ± 2.5 cm. The lsSMA-rsSMV distance was measured in 76 (95%) patients, and the mean distance was 2.21 ± 0.6 cm (Table 2).

**Table 2:** Results

	n (%)
ICV and ICA roots both located	74 (92.5%)
ICA located in the front of the ICV	43 (58.1%)
ICA located behind the ICV	31 (41.9%)
Accuracy in predicting the relationship between the ICA and ICV	72 (97.2%)
Location of the GTH relative to the pancreas was determined	75 (93.7%)
Accuracy in predicting the GTH location	73 (97.33%)
MCA roots located	47 (58.75%)
Accuracy in predicting the MCA location	37 (78.72%)
MCV roots located	51 (63.75%)
Accuracy in predicting the MCV location	43 (84.31%)
ICV-GTH distance (mean ± SD)	4.28 ± 2.5 cm
lsSMA-rsSMV distance (mean ± SD)	2.21 ± 0.6 cm

## Discussion

Right hemicolectomy can usually be performed through an open or laparoscopic approach. Some studies have shown that laparoscopic right hemicolectomy has some advantages over open hemicolectomy [21–23], but laparoscopic surgery requires a longer operative time and needs more practise [21, 24]. Therefore, it is necessary to understand the anatomical structure of and relationships within the operation area before laparoscopic right hemicolectomy, especially when performed with the CME procedure.

To perform right hemicolectomy with a central approach, surgeons usually need to find the ileocolic vessels first, separate the ICV and ICA roots and then ligate them separately.

When performing CME with CVL, ligations of the ICV and right colic vein (if present) are performed along the right side of the SMV. When the cephalad side is dissected, the GTH will be encountered in most patients. In cases of unclear anatomy, intraoperative bleeding mostly results from damage to these vessels, especially the ICV and GTH, which might lead to massive bleeding.

The left side of the SMA is the boundary in CME operations, and only when this margin is reached can sufficient lymph node dissection be achieved. However, in patients with a high BMI, it is difficult to determine the boundary because the main blood vessels are usually covered by thickened mesenteric adipose tissue.

As mentioned above, the key points of CME with CVL are to locate the roots of the ICV, ICA and GTH, the right side of the SMV and the left side of the SMA. Previous studies have reported CT-based vascular prediction techniques, but these lack accuracy comparisons [25] or require additional analysis techniques, such as CT angiography or colonography [26]. This study evaluated the accuracy of MSCT with coronal reconstruction in predicting critical anatomical sites, and this approach reduced the technical difficulties when used in practice.

Portal phase MSCT data were used for coronal reconstruction because these data can simultaneously show the central veins and arteries for right hemicolectomy, including the ICV, ICA, GTH, SMV and SMA. Preoperative visualisation of these vessels can be used to determine the extent of the CVL region during right hemicolectomy, whilst postoperative visualisation of these vessels can be used to assess the quality of CME [27].

**Fig. 1:**
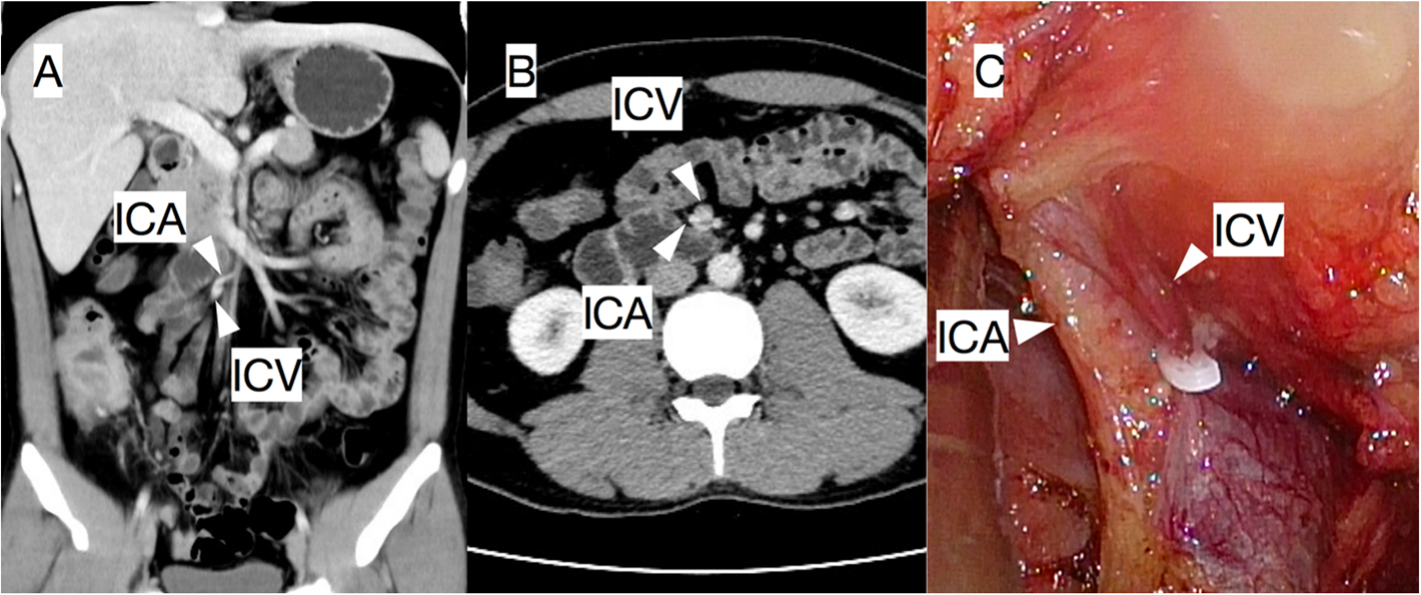
In a 34-year-old male patient whose ICA was behind the ICV, the anatomical relationship between the ICA and ICV was determined preoperatively by cross analysing the coronal (**A**) and axial images (**B**), and this relationship was confirmed by the intraoperative findings (**C**)

To determine the anatomical relationship between the ICV and ICA, as well as analyse the coronal reconstructed images, it is necessary to simultaneously examine both vessels on the axial images through the software’s (Philips InteliSpace Portal) positioning function to increase the accuracy (Fig. 1).

Because of the high accuracy in predicting the location of the ICV and ICA and the relationship between these vessels, this technique might be helpful in the initial stages of right hemicolectomy performed through the central approach. For example, if the ICA crosses behind the ICV, ligation and dissection of the ICA root must be carried out behind the SMV.

**Fig. 2:**
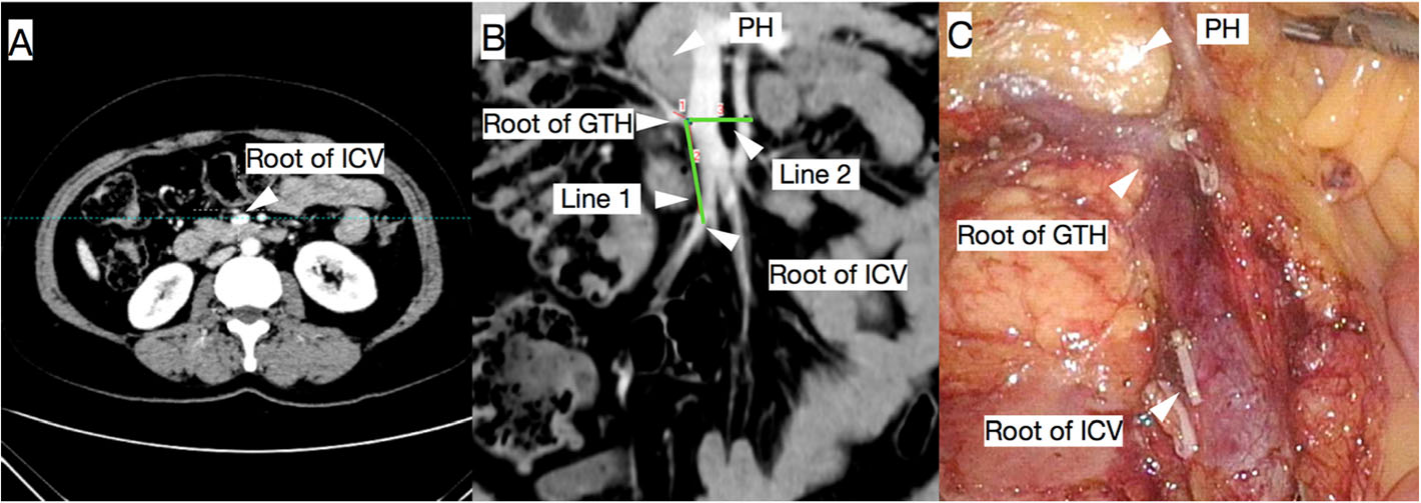
By analysing the axial (**A**) and coronal (**B**) MSCT images of a 49-year-old female patient, MSCT with coronal reconstruction was able to help locate the roots of the GTH and ICV, find the location of the pancreas head (PH) and measure the ICV-GTH distance (line 1). The anatomical relationship between these structures could be used for guidance during surgery (**C**)

In this study, MSCT with coronal reconstruction located the GTH in 93.7% of patients, and the accuracy was 97.33%. After locating the roots of the ICV and GTH, the straight-line distance from the ICV root to the GTH root was also able to be measured with this technique (Fig. 2). These findings could help surgeons predict the operation route along the SMV and might reduce the occurrence of intraoperative vascular injury and bleeding [12].

**Fig. 3:**
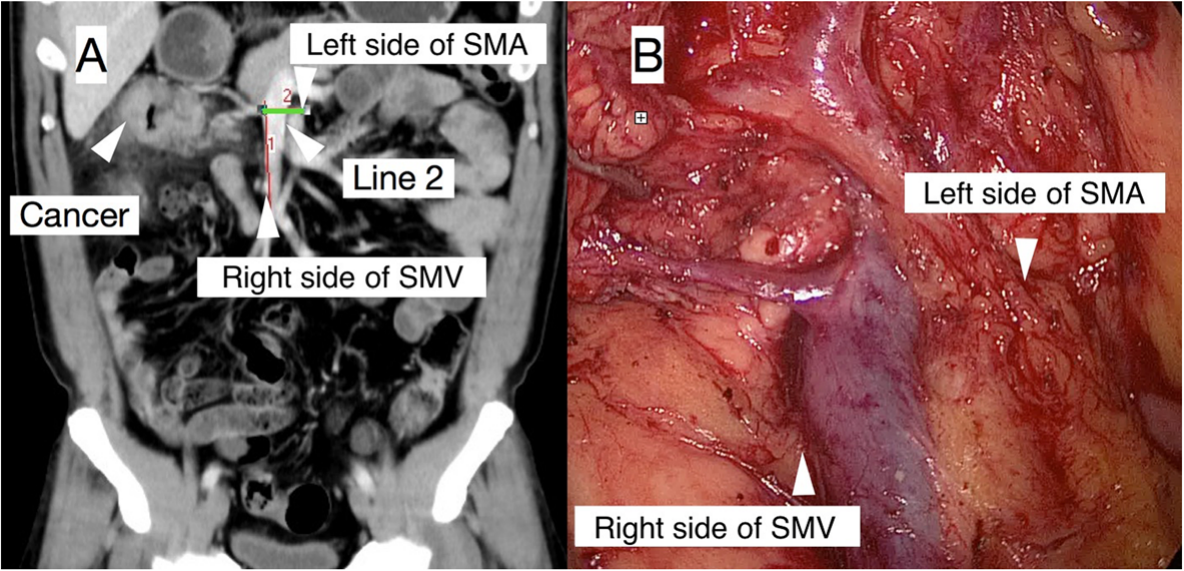
In a 42-year-old patient with hepatic flexure cancer, the left side of the SMA and the right side of the SMV were located on the coronal reconstruction (**A**), and the lsSMA-rsSMV distance (line 2) was measured, which was confirmed by the intraoperative findings (**B**)

Furthermore, by analysing the coronal reconstructed images, this technique could help locate the lsSMA and rsSMV and then measure the maximum distance between them (Fig. 3). Typically, when performing CME with CVL, dissection along the left side of the SMA is required, but locating this side during the operation can be challenging in some patients with a high BMI. As shown below, reconstructed coronal MSCT images could help to predict the border. By measuring the lsSMA-rsSMV and ICV-GTH distances; it was possible to estimate the CVL region size.

These measurements may be difficult to translate to the operative field of a laparoscopic procedure, and more studies are needed. The usefulness of these measurements in surgery differs amongst patients and surgeons. However, according to our experience, these measurements might be helpful in patients whose SMVs cannot be located at first glance during laparoscopic surgery.

**Fig. 4:**
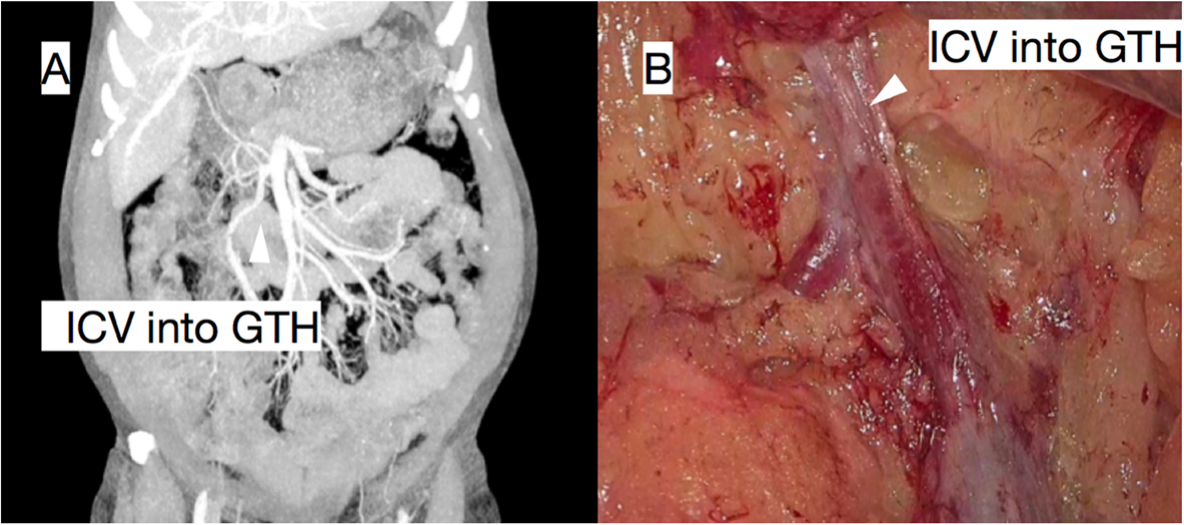
In a 62-year-old male patient with proximal transverse colon cancer, the maximum intensity projection (MIP) reconstruction of the coronal plane (**A**) and intraoperative images (**B**) showed that the ICV directly flowed into the GTH

Furthermore, coronal reconstruction might help surgeons detect important vascular and anatomical variations preoperatively, as shown in the following figure (Fig. 4), in which a patient’s ICV flows directly into the GTH and, if it went unnoticed, may cause intraoperative injury or bleeding.

Compared with CT angiography, MSCT with coronal reconstruction conveys more information of perivascular anatomical sites, such as the pancreas, duodenum, tumour and lymph nodes. The relationship between these tissues can guide CME with CVL.

With the predicted information mentioned above, surgeons might be able to individualise the operation plan, predict the focus of the surgery and develop a more appropriate path, which might improve the efficiency of the operation and completion rate of CME and reduce the risk of intraoperative injury or bleeding. Of course, this still needs to be demonstrated by prospective studies.

This technology is also cost-effective. Patients only need conventional abdominal contrast-enhanced MSCT scans without additional CT angiography, making this technology more suitable for clinical application, especially for hospitals restricted in equipment or technology and for patients who are afraid of increasing the radiation dose they receive.

However, this study also found that this technique was not suitable for locating the middle colic vessels due to a low detection rate, mainly because these vessels were too small to be detected by conventional enhanced MSCT scans.

In addition, the right colic vessels and superior right colic vessels were not included in this study because if the right side of the SMV and the roots of the ICV and GTH were successfully located, it would relatively be easy to find the right colic vessels and superior right colic vessels when dissecting along the right side of the SMV and between the roots.

## Conclusions

MSCT with coronal reconstruction can locate the roots of the ICV, ICA and GTH, predict the SMA and SMV boundaries, enable analysis of the anatomical relationship between these vessels and estimate the range and size of the CVL region. MSCT has limited detection of the vascular anatomy of the middle colic vessels. With its satisfactory accuracy in predicting and visualising information of key anatomical sites, this technology is of some predictive value for CME with CVL in right hemicolectomy. We recommend that this technique be performed as a routine preoperative workup, if possible.

## Data Availability

All data generated or analysed during this study are included in this published article.

## Abbreviations

CME: Complete mesocolic excision
CVL: Central vascular ligation
MSCT: Multi-slice spiral computed tomography
ICV: Ileocolic vein
ICA: Ileocolic artery
GTH: Gastrocolic trunk of Henle
ICV-GTH distance: Distance from the ICV root to the GTH root
lsSMA-rsSMV distance: Maximum distance from the left side of the superior mesenteric artery to the right side of the superior mesenteric vein
PH: Pancreas head
MDCT: Multidetector computed tomography
3D-CT: Three-dimensional CT
BMI: Body mass index
